# Postoperative outcomes of minimally invasive adrenalectomy: do body mass index and tumor size matter? A single-center experience

**DOI:** 10.1186/s12893-022-01725-6

**Published:** 2022-07-19

**Authors:** Felipe Girón, Carlos Eduardo Rey Chaves, Lina Rodríguez, Roberto Javier Rueda-Esteban, Ricardo E. Núñez-Rocha, Sara Toledo, Danny Conde, Juan David Hernández, Marco Vanegas, Ricardo Nassar

**Affiliations:** 1 Department of Surgery, Fundación Santa Fé de Bogotá, 110111 Bogotá, DC Colombia; 2 School of Medicine, Universidad del Rosario, Carrera 7 # 117-15, 111711 Bogotá, DC Colombia; 3 School of Medicine, Universidad de los Andes, 111711 Bogotá, DC Colombia; 4 Fundación Santa Fé de Bogotá, 110111 Bogotá, DC Colombia

**Keywords:** Laparoscopic, Adrenalectomy, Hypertension, Robot-assisted, Outcomes

## Abstract

**Background:**

Since Gagner performed the first laparoscopic adrenalectomy in 1992, laparoscopy has become the gold-standard procedure in the treatment of adrenal surgical diseases. A review of the literature indicates that the rate of intra- and postoperative complications are not negligible. This study aims to describe the single-center experience of adrenalectomies; and explore the associations between body mass index (BMI) and tumor volume in main postoperative outcomes.

**Methods:**

Retrospective observational study with a prospective database in which we described patients who underwent adrenalectomy between January 2015 and December 2020. Operative time, intraoperative blood loss, conversion rate, complications, length of hospital stay, and comparison of the number of antihypertensive drugs used before and after surgery were analyzed. Analysis of BMI and tumor volume with postoperative outcomes such as anti-hypertensive change (AHC) in drug usage and pre-operative conditions were performed.

**Results:**

Forty-five adrenalectomies were performed, and all of them were carried out laparoscopically. Four were performed as a robot-assisted laparoscopy approach. Nineteen were women and 26 were men. Mean age was 54.9 ± 13.8 years. Mean tumor volume was 95.698 mm^3^ (3.75–1010.87). Mean operative time was shorter in right tumors (2.64 ± 0.75 h) than in left tumors (3.33 ± 2.73 h). Pearson correlation was performed to assess the relationship between BMI and AHC showing a direct relationship between increased BMI and higher change in anti-hypertensive drug usage at postoperative period r(45) = 0.92, p > 0.05 CI 95%. Higher tumor volume showed a longer operative time, r(45) = 0.6 (p = 0.000 CI 95%).

**Conclusions:**

Obese patients could have an increased impact with surgery with an increased change in postoperative anti-hypertensive management. Tumor volume is associated with increased operative time and blood loss, our data suggest that it could be associated with increased rates of morbidity. However, further prospective studies with larger sample sizes are needed to validate our results.

## Background

Since 1992, Gagner et al. described the first laparoscopic adrenalectomy (LA) [1, 2], advances in technique and multidisciplinary approach allow for treatment in the present time adrenal masses up to 11–12 cm with acceptable morbidity rates that could vary between 0 and 27% in some series of cases [2–6]. Traditionally, LA it’s considered the gold standard approach to treating adrenal masses due to the lower rates of morbidity, mortality, decreased in-hospital stay, and lower intraoperative blood loss. Nevertheless, an open approach should be preferred in selected cases of large tumors with suspected or confirmed malignancy and compromise of surrounding organs [2, 7–10].

Minimally invasive techniques are now arising in metabolic surgery such as the retroperitoneoscopic approach; Walz et al. [11] reported safe results in comparison with open or laparoscopic techniques. Since the advancement of robotic technology in surgery, novel approaches have been described for abdominal procedures including metabolic and gastrointestinal surgery, and the field of adrenalectomies isn’t an exception [12]. In 1999 Piazza et al. [13] reported the first adrenalectomy with robotic technology, since then, multiple authors have published their results such as Ragavan and Vatansever et al. [6, 14] showing improvement in postoperative outcomes in terms of lesser complications rate, conversion rate, and shorter in-hospital stay compared with conventional laparoscopic approach [14].

Indications for adrenalectomy are broad [15]. These indications include (1) functional adrenal tumors regarding of the size (Cushing’s syndrome, Conn’s syndrome, Pheochromocytomas), (2) malignancy suspicious or malignant tumor (adrenocortical cancer, malignant pheochromocytoma, metastatic tumors) and (3) nonfunctional tumors with malignancy risk located one-sided or bilateral [15]. Regarding the clinical conditions associated with adrenal masses, guidelines have established that adrenal masses that lead to primary aldosteronism (PA) are a common cause of secondary hypertension, accounting for more than 10% of patients with hypertension. A small fraction of patients with PA secondary to adenomas can also develop hypercortisolism secretion that intensifies the adverse effects of aldosteronism and heighten hypertension, hypokalemic alkalosis, impaired glucose tolerance, and increase the cardiovascular risk [16, 17].

In surgery, risk factors analysis is a cornerstone for decreasing postoperative complications; in metabolic surgery, this is not an exception. Multiple authors tried to evaluate the influence of BMI or tumor volume on the postoperative outcomes, and in cases of adrenalectomies, drug usage and blood pressure changes after surgery are frequently used as the main outcomes [18, 19]. However, this topic is still a matter of research. The aim of this study is to describe a single-center experience of adrenalectomies by describing demographics, surgical characteristics, and outcomes. Also, to explore the associations between BMI and tumor volume in main postoperative outcomes.

## Methods

Following Institutional Review Board approval, all patients over 18 years old who underwent adrenalectomy between January 2015 and December 2020, were registered. The present study was performed in a single reference center, by a single metabolic/endocrine surgeon. A retrospective analysis of patients who complied with inclusion criteria was made for the study. Ethical compliance with the Helsinki Declaration, current legislation on research Res. 008430-1993 and Res. 2378-2008 (Colombia), and the International Committee of Medical Journal Editors (ICMJE) were ensured under our Ethics and Research Institutional Committee (IRB) approval.

Preoperative data included patients' demographics, clinical and blood pressure history. Procedure approach, laterality, tumor volume, and blood loss were included in the intraoperative data. Postoperative data included early and late complications and a comparison between antihypertensive drugs needed before and after surgery.

Descriptive statistics of all study parameters were provided. Data were analyzed using IBM SPSS Statistics 25 software. Continuous data were summarized by their mean, standard deviation, median, minimum, and maximum. Categorical data were summarized by their frequency and proportion. Bivariate analysis was performed to explore the association between BMI, and tumor volume with the main postoperative outcome (change in anti-hypertensive drug) and pre-operative features (systolic hypertensive crisis defined as systolic blood pressure > 180 mmHg). Qualitative variables were analyzed using Chi-square statistics (Fisher's exact test when appropriate). Quantitative variables were analyzed, based on normality, with Spearman's or Pearson's associations correlation coefficients accordingly. P-value < 0.05 was accepted as statistically significant.

## Results

### Preoperative characteristics

A total of 45 patients underwent adrenalectomy. Male patients constituted 57.78% (n = 26). Mean age was 54.9 ± 13.82 years. Mean BMI was 26.94 ± 4.59 kg/m^2^. History of hypertension was presented in 91.1% and T2DM in 5 patients. Previous episodes of hypertensive crisis were present in 15.56% of the patients (n = 7). In terms of previous anti-hypertensive drugs, preoperatively, patients use a mean of 1.8 ± 1.2 (range 0–5) medications. Primary hyperaldosteronism was the most frequent indication for surgery in 31.8% of the cases (n = 14) followed by adrenal masses (25% n = 11); malignancy was suspected in 2 cases (metastatic disease and primary adrenal mass), multiple endocrine neoplasia type 2 was observed in 1 case. Summarized characteristics are displayed in Table 1.

**Table 1:** Demographic and clinical characteristics

Variable	Result
Demographics	Mean (SD)
Age	54.9 (13.8)
Body mass index	26.94 (4.5)

Gender	% (n)
Male	57.7 (26)
Female	42.2 (19)

	% (n)
Previous history	
Hypertension	91.1 (41)
Type 2 Diabetes Mellitus	11.1 (5)
Hypertensive crisis (> 180 mmHg)	15.5 (7)
American Society of Anesthesiologists Classification	
ASA I	2.2 (1)
ASA II	28.8 (13)
ASA III	66.6 (30)
ASA IV	2.2 (1)
Indication for surgery	
Primary hyperaldosteronism	31.8(14)
Adrenal adenoma	11.3 (5)
Hormone active adrenal tumor	4.5 (2)
Pheochromocytoma suspicion	13.6 (6)
Adrenal mass	25 (11)
Cushing's	4.5 (2)
Undetermined adrenal mass	2.2 (1)
Adrenal metastases	2.2 (1)
Benign tumor	2.2 (1)
MEN-2A due to RET mutation	2.2 (1)
Malignancy suspicion	2.2 (1)

### Surgical characteristics

The laparoscopic approach was preferred in most of the cases (91.1%, n = 41) followed by robotic adrenalectomy. Conversion to open procedure was required in 1 patient. The mean operative time was 3.1 ± 2.13 h. Mean operative time was shorter in right tumors (2.64 ± 0.75 h) than in left tumors (3.33 ± 2.73 h). Intraoperative blood loss mean was 66.6 ± 134.7 cc. Tumor volume was calculated according to imagenological findings (Width × Lengthy × Thickness); mean tumor volume was 95.69 ± 203.02 cubic centimeters (range 3.75–1060). Right-sided adrenalectomy was performed in 44.4% of the cases; a bilateral approach was required in 3 cases. Summarized characteristics are displayed in Table 2.

**Table 2:** Surgical characteristics

Variable	Results
Surgical approach	% (n)
Laparoscopic	91.1 (41)
Robotic	9.09 (4)
Conversion	2.2 (1)

Intraoperative characteristics	Value (SD)
Operative time	3.1 h (2.13)
Blood loss	66.6 cc (134.7)
Tumor volume	95.6 cc3 (203.2)

Side of surgery	% (n)
Right	44.4 (19)
Left	48.89 (21)
Bilateral	6.8 (4)

### Outcomes

The postoperative outcomes are summarized in Table 3. The mean in-hospital stay was 5.2 ± 4.5 days. No patient needed an intensive care unit stay. At 30 days of follow-up, the mortality rate was 0%, morbidity was observed in 26.6% (n = 12) of the cases and were classified according to the Clavien-Dindo classification (Type 1 = 16%; Type 2 = 84%). Patients with Clavien-Dindo Type 1 required electrolytic replacement, and Type 2 patients required blood transfusions and diuretic usage. The re-admission rate was 6.67% (n = 3).

**Table 3:** Outcomes

Variable	Result
Postoperative outcomes	Mean (SD)
In-Hospital stay	5.2 (4.5)

	% (n)
Mortality	0% (0)
Morbidity	26.6 (12)
Re-admission	6.6 (3)
Clavien Dindo	
Type 0	73.3 (33)
Type 1	4.4 (2)
Type 2	10 (22.2)
Pathology analysis	
Hyperplasia	4.4 (2)
Adenoma	42.2 (19)
Pheocromocytoma	17.78 (8)
Myelolipoma	4.4 (2)
Cyst	2.2 (1)
Metastases	4.4 (2)
Cortical Hyperplasia	13.3 (6)
Ganglioneuroma	2.2 (1)
Normal Adrenal Gland	2.2 (1)
Bronchogenic cyst	2.2 (1)
Medular Hyperplasia	2.2 (1)
Neoplasm	2.2 (1)

Drug ussage	Mean (SD)
Pre-operative	1.8 (1.2)
Postoperative	0.73 (0.96)
Change (AHC)	1.06 (1.1)

Pathology analysis shows that most of the cases were adenomas (42.2% n = 19), followed by pheochromocytoma in 17.7% of the cases (n = 8). Mean of anti-hypertensive drug usage during the postoperative time was 0.73 ± 0.96 medications (range 0–3). Comparison of drug usage in the pre-operative and postoperative periods shows a mean change (AHC) of 1.06 ± 1.15 anti-hypertensive medications. Comparison between RA and LA shows an increased rate of morbidity favoring robotic adrenalectomy (RA = 0%; LA = 32.25%).

### Statistical analysis

Associations between pre-operative characteristics, outcomes, and AHC during the pre/post-operative period were performed. Patients with pre-operative systolic hypertensive crisis show a bigger AHC with a statistically significant value (p < 0.05, CI 95%). Pearson correlation was run to assess the relationship between BMI and AHC; there was a small correlation between increased BMI and higher change in anti-hypertensive drugs at postoperative period r(45) = 0.92, p > 0.05 CI 95% showing a statistical relationship.

**Fig. 1:**
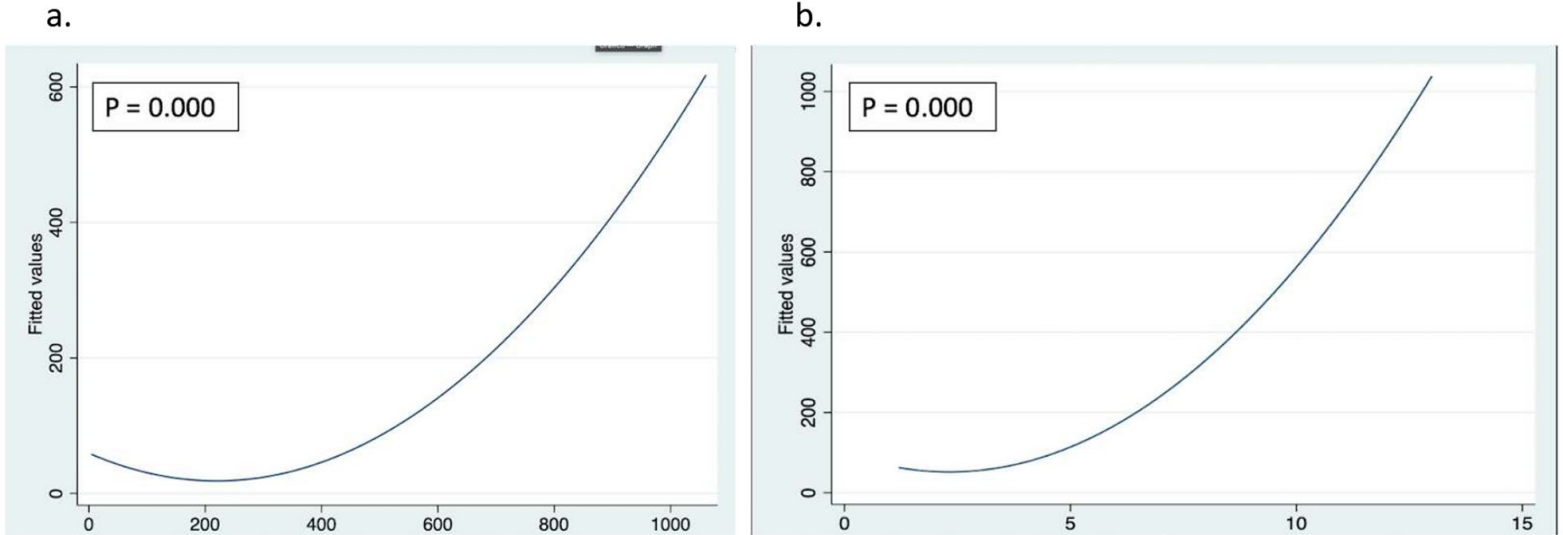
Scatter fitted plot: **a** Intraoperative blood loss vs tumor volume and **b** operative time vs tumor volume. Pearson correlated test

Tumor volume was evaluated to explore possible associations with postoperative outcomes. As well, a Pearson correlation was performed between tumor volume and AHC and showed that there is a negative small correlation between increased tumor volume and change in anti-hypertensive drugs in postoperative period r(45) = − 0.10. However, it failed to establish statistical relationship (p = 0.5). Furthermore, tumor volume in Pearson correlation showed an increased correlation in intraoperative blood loss r(45) = 0.6, with a statistically significant value (p = 0.000 CI 95%). In terms of operative time, tumor volume showed to be related to higher operative times, r(45) = 0.6 with a statistically significant value (p = 0.000 CI 95%) (see Fig. 1). Tumor volume and morbidity failed to reach a statistically significant association (p = 0.08 CI 95%). Differences in operative time and side of surgery failed to show a statistical relationship. Summarized data are shown in Table 4.

**Table 4:** Statistical analysis

Variable	AHC p value (Pearson correlation)	Hypertensive Crisis p value
BMI	> 0.05 (0.29)	> 0.05
Tumor volume	0.50 (− 0.10)	0.84

## Discussion

Minimally invasive techniques for adrenalectomy such as RA or LA approaches are increasing their usage for over 20 years since the first description of robotic adrenalectomy [11, 13, 20]. In the present day, LA is considered the gold standard approach to treating adrenal masses, due to lower rates of morbidity, mortality, decreased in-hospital stay, and lower intraoperative blood loss compared with the open approach [2]. Nonetheless, the data reported is heterogeneous in terms of perioperative characteristics or postoperative outcomes [2].

LA and RA have shown, in several studies, the safety and feasibility of these procedures with an acceptable complications rate [2, 21]. Actual literature shows a morbidity rate that varies between 0 to 27% in RA, and in most cases corresponding to Clavien-Dindo 1 or 2 [22, 23]. In our population, the general morbidity rate was 26%, and patients that followed robotic surgery show a lesser rate of complications compared to the laparoscopic approach (33.3% vs 0%). It is important to declare, that morbidities were only typed 1 or 2 according to Clavien-Dindo classification, data that is comparable with the previously reported literature [23].

Conversion rate is another important topic when analyzing minimally invasive techniques and the previous series shows a rate between 0 and 40% [24–26]. In our population, conversion to open surgery was 2.2% and was one of the laparoscopic procedures data that it’s similar to the worldwide literature (26). Additionally, the operative time it’s a matter of debate because not all the studies report cofounding factors for the preparation time (robot disposition, trocar’s location, anesthesia, docking time), and for that reason, this variable could be biased [2, 27]. Nonetheless, some series report a variable time between 89 and 215 min [2, 28]. In our population, the mean operative time was 180 min with a variable range between 60 and 420 min, closer to the one reported by Materazzi et al. [2].

Another important factor of minimally invasive techniques is the total length of hospitalization; a comparison between LA and RA in larger studies trends to favor RA with a lesser in-hospital stay, with a statistically significant value according to Vatansever et al. [14] who reported this in a retrospective case–control study of 1005 patients. Our data support these findings, RA mean of hospital stay was significantly lesser compared with LA (3 vs 5.4 days). However, failed to reach a statistical relationship, probably due to the small sample size.

Obesity has become a worldwide concern highly related to increased morbidity and mortality [19]. The association between increased BMI and operative and postoperative complications it’s still a matter of research [19]. Some authors, such as Danwang et al. [19] suggest that postoperative outcomes do not increase in obese patients. However, studies reported are limited [19, 29–32]. In our population, increased BMI is not related to conversion rate, morbidity, or readmission rates, supporting the actual data. Nevertheless, our data show a relationship between BMI and changes in postoperative anti-hypertensive medication as well as the systolic hypertensive crisis before surgery, suggesting that obese patients usually need more pre-operative medication, and surgery has an increased impact on this group of patients, similar to the results reported by Van Der Linde et al. [33].

Tumor size also, it’s a clinical finding that could change the approach-decision making. This happens because, usually, in tumors larger than 5 cms, malignancy should be suspected [6]. In these cases, an open approach could be preferred [6], and frequently, tumors > 5 cm are related to increased operative time and intraoperative blood loss, according to Morelli et al. [34]. Our data support these findings because tumor volume shows a linear relationship with blood loss and operative time. Notwithstanding, in contrast with other studies [33, 34], tumor size impact on morbidity was not documented in our study, this could be related to the small sample size.

Among the limitations of our study are the retrospective nature, and small sample size. However, the strengths of our study are that our data increases the literature in terms of minimally invasive techniques for adrenalectomy, and in contrast with other studies, support that actually sizes does matter in terms of operative time and intraoperative blood loss. Although in our study there isn’t a relationship between tumor volume and morbidity, the p-value suggests that probably with an increased sample size data can reach a statistically significant value. Also, our data suggest that obese patients could have an increased benefit with surgery, in terms of antihypertensive drug usage.

## Conclusions

Minimally invasive techniques such as RA or LA are safe, and feasible approaches, with acceptable rates of morbidity and hospital length. Obese patients seem to have an increased impact in terms of postoperative anti-hypertensive medication reduction. Tumor volume is associated with increased operative time and blood loss, our data suggest that this could be associated with increased rates of morbidity. However, further prospective studies with larger sample sizes are needed to prove our results.

## Data Availability

The datasets used and/or analyzed during the current study are available from the corresponding author upon reasonable request.

## Abbreviations

BMI: Body mass index
AHC: Anti-hypertensive change
LA: Laparoscopic adrenalectomy
RA: Robotic adrenalectomy
PA: Primary aldosteronism
ICMJE: International Committee of Medical Journal Editors
IRB: Ethics and Research Institutional Committee
T2DM: Type 2 diabetes mellitus
